# ProCarbDB: a database of carbohydrate-binding proteins

**DOI:** 10.1093/nar/gkz860

**Published:** 2019-10-10

**Authors:** Liviu Copoiu, Pedro H M Torres, David B Ascher, Tom L Blundell, Sony Malhotra

**Affiliations:** 1 Department of Biochemistry, University of Cambridge, Tennis Court Road, Cambridge, CB2 1GA, UK; 2 Department of Biochemistry, University of Melbourne, Flemington Road, Parkville, Australia

## Abstract

Carbohydrate-binding proteins play crucial roles across all organisms and viruses. The complexity of carbohydrate structures, together with inconsistencies in how their 3D structures are reported, has led to difficulties in characterizing the protein–carbohydrate interfaces. In order to better understand protein–carbohydrate interactions, we have developed an open-access database, ProCarbDB, which, unlike the Protein Data Bank (PDB), clearly distinguishes between the complete carbohydrate ligands and their monomeric units. ProCarbDB is a comprehensive database containing over 5200 3D X-ray crystal structures of protein–carbohydrate complexes. In ProCarbDB, the complete carbohydrate ligands are annotated and all their interactions are displayed. Users can also select any protein residue in the proximity of the ligand to inspect its interactions with the carbohydrate ligand and with other neighbouring protein residues. Where available, additional curated information on the binding affinity of the complex and the effects of mutations on the binding have also been provided in the database. We believe that ProCarbDB will be an invaluable resource for understanding protein–carbohydrate interfaces. The ProCarbDB web server is freely available at http://www.procarbdb.science/procarb.

## INTRODUCTION

Carbohydrates are amongst the most versatile classes of ligands, being able to form complex, branched glycans from monosaccharide units. This generates a complex structural pattern, commonly referred to as the glycocode, which carbohydrate-binding proteins are able to decipher (1). These proteins are known to play important roles in many cellular processes, including embryogenesis (2), immune response (3), protein trafficking (4), bacterial-toxin uptake (5) and viral infection (6). However, protein–carbohydrate interfaces are not well characterized, which is partly a consequence of the absence of a standardized nomenclature for sugars. Moreover, identifying sugar moieties in the Protein Data Bank (PDB) (7) is challenging, as some of the carbohydrate entries are poorly annotated (8). This is in part due to the large number of naturally occurring monosaccharides, but also due to the multiple ways saccharide units may be linked and the complex branching capacity of polysaccharides.

In the present PDB format, the distinction between the carbohydrate ligand and its saccharide units is not trivial. Hence, interactions cannot be computed without using protein structure visualization software such as PyMol (9) and Chimera (10). This has hindered efforts to characterize systematically and to understand the underlying molecular features of protein–carbohydrate interfaces. Another limitation of current online resources that attempt to decipher the 3D architecture of carbohydrate ligands, such as pdb-care (11), is that they do not differentiate between the covalently bound carbohydrates (post-translational modifications), crystallographic errors (broken ligands) and true, complete ligands.

Due to these restraints, it is non-trivial to incorporate relevant biological information (such as biophysical measurements, interface interactions, the structure of the ligand and mutagenesis analysis) of protein–carbohydrate complexes into databases. Protein–carbohydrate complexes are poorly represented in databases such as Platinum (12) (5.4%), PDBbind (13) (6%) and MOAD (14) (8%), which collect ligand-binding affinity data for proteins. This is due to experimental difficulties encountered while working with carbohydrates, including their low affinity values but high ligand specificity, and their being part of more complex biological molecules, such as gangliosides, which contain functional groups other than sugars (15–17). Furthermore, none of the above-mentioned repositories provides information on protein–carbohydrate interfaces. The scarcity of available protein–carbohydrate datasets, some of which do not distinguish between the whole ligand and its units, has limited the applicability and accuracy of methods developed to investigate protein–carbohydrate interactions (18–20). Recently, there have been efforts to create highly curated and specific structural repositories for glycan-binding proteins. Unilectin3D (21) hosts experimentally solved structures for lectins, across all kingdoms (including viruses) generating both SNFG (Symbol Nomenclature for Glycans) (22) depictions and IUPAC (International Union of Pure and Applied Chemistry) (23) notations. Carbohydrate-active enzymes are extensively covered in CaZy (Carbohydrate-active enzyme) database (24), and recently they have mapped 3D structures from PDB to their enzyme nomenclature, identifying over 100 types of carbohydrate-like molecules as biological relevant ligands. Another useful online resource for glycan structures and motifs is GlyTouCan (25), which hosts over 100 000 structures and identifies 800 monosaccharides. Resources combining structural information with prediction tools, mass spectrometry and NMR data have also been developed in recent years: ProGlycProt V2.0 (26), for prokaryotic glycoproteins and glycosyltransferases, Carbohydrate Structure Database (27), for bacteria, archaea, fungi and plants, and Glyco3D (28), for a general overview on glycan binding proteins ranging from glycosaminoglycan-binding proteins to antibodies.

Here we describe ProCarbDB, a freely accessible, user friendly database that comprises of 5242 true protein–carbohydrate complexes. For a given PDB entry, ProCarbDB correctly annotates and displays the complete carbohydrate ligand present, the ligand interactions and binding affinities (where available), and the effects of experimentally validated mutations on the binding affinity. We believe that ProCarbDB will be an invaluable resource for understanding the features of protein–carbohydrate interfaces and their recognition patterns. It will also facilitate the development of structure-based machine-learning algorithms that can be trained to predict the binding affinity between a putative carbohydrate-binding protein and its saccharide ligand.

## MATERIALS AND METHODS

### Data acquisition and inclusion criteria

An exhaustive list of PDB ligands classified as carbohydrates was obtained using a stand-alone copy of pdb-care (11) and manually curating the results. We obtained a list of 900 carbohydrate PDB Ligand IDs. We retrieved around 13 000 X-ray crystal structures containing at least one saccharide moiety (for the complete pipeline flowchart see Supplementary Figure S1). In comparison, PDB annotates <600 molecules as saccharides.

Using a graph-based approach, we filtered out the possible true negatives:

- Structures that contain only post-translational modifications (such as N/O-linked glycosylation).
- Structures where no sugar ligand was in the proximity of a protein chain (at least one atom of the ligand has to be 4Å or closer to any heavy atom of a protein residue).
- Structures where no protein chain was longer than 30 amino acids (Supplementary Figure S1).
- Structures that contained only crystallographic adjuvants (e.g. B-octylglucoside) by using a semi-automatic text-mining algorithm based on cross-reference between well-established databases such as UniProt (29), PDB (7) and ENZYME database (30).

As a result of this filtering approach, we obtained 5242 protein–carbohydrate complexes. It is important to note that several amphipathic molecules (BOG, DA8, DEG, KGM etc.), which are usually used as, or are very similar to, detergents, are actually true biological ligands in a number of entries, such as 1UWF and 2G3N.

### Ligand sanitization

Using the above-mentioned graph-based approach and the CONNECT records of the PDB file, we first checked the integrity of the ligands by determining the saccharide units that constitute the whole ligand. Next, we calculated distances from terminal atoms of the ligands (i.e. atoms that only have one covalent bond) to all other atoms. For some entries the distance was within the range expected for a covalent bond, but not listed in the CONNECT records. This resulted from either: (i) overlapping of residues due to the presence of stereoisomers in the crystallization solution (e.g. PDB ID: 5MTU) or (ii) broken ligands (e.g. PDB ID: 5TPC). To solve the former issue, we used the occupancy register in the PDB structure dictionary, where if the total occupancy of both units is equal to 1 they are overlapping. To solve the second issue, before generating a new bond we ensured that no superposed atoms were present and that valence rules were maintained. By using these methods, we were able to identify not only pure carbohydrate ligands but also glycoconjugates, such as PDB ID:2JDH.

The ligands are presented in a table along with their 3D representation, in which PDB Ligand IDs are coloured according to the SNFG nomenclature (22) (Supplementary Figure S2). Furthermore, we also generate IUPAC or LINUCS (Linear Notation for Unique description of Carbohydrate Sequences) (31) notations where possible.

### External resources

We mapped these crystal structures with biophysical measurements using two available databases: PDBbind (13) and MOAD (14). Using a series of text mining and request functions, we were able to link 967 protein–carbohydrate complexes with an affinity value. Furthermore, using a combination of APIs from PDB and UniProt, we are able to provide users direct mappings to other well-established databases like UniProt, Pfam (32) and enzyme commission number. In addition, curated mutagenesis information for the protein–carbohydrate complexes present in the database is being continuously added manually.

### Database architecture and web interface

The database architecture (Supplementary Figure S3) was written using the SQLAlchemy Python (version 2.7.1). All data are stored in a PostgreSQL server. For World Wide Web Connectivity, the Flask Python module (version 1.0.2) was used.

The website is written in HTML5 using CSS, Javascript and JQuery as well as a Bootstrap (version 4) framework. JINJA2 templating language for Python was used to dynamically generate HTML templates. All 3D rendering is done using NGL (33).

The database website is freely available at: http://www.procarbdb.science/procarb/.

## RESULTS: DATABASE FEATURES

### Web interface

The access point for documentation, resources, data and visualization methods is http://www.procarbdb.science/procarb/. The documentation can be accessed using the ‘Help’ page from the navigation tab (Supplementary Figure S4). Links to specific sections of the ‘Help’ page are also provided based on the user’s current location on the website.

**Figure 1.**
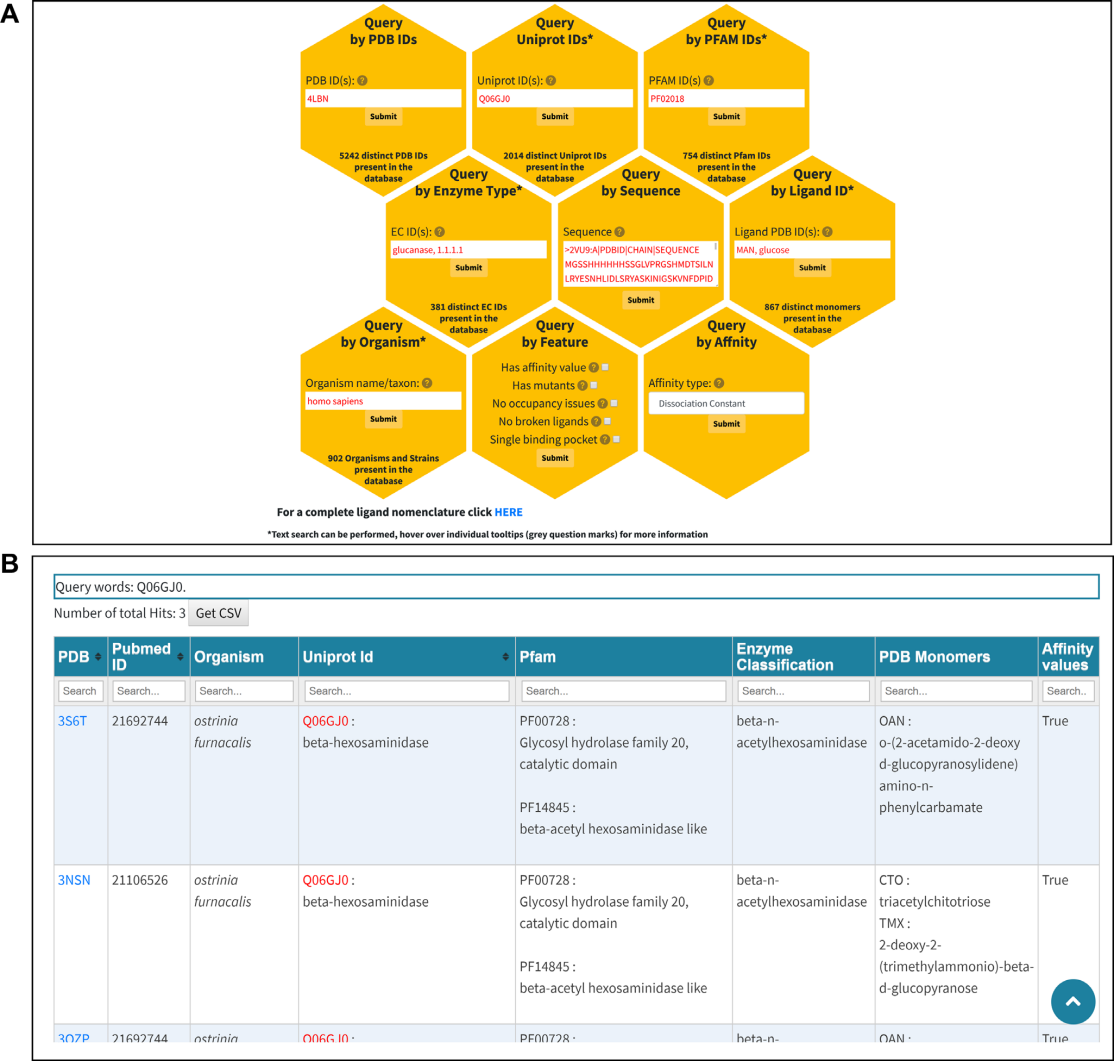
ProCarbDB web interface search and results pages. (**A**) ProCarbDB offers nine query modes, with detailed information available about each query type through the ‘Help’ page at the top navigation bar and through on-page help in the form of question mark tooltips. Fields marked with ‘*' support text search. At the bottom of this page, we offer a link that will display all the PDB Ligand IDs present in ProCarbDB along with PDB-derived name, IUPAC nomenclature and InChIKey. (**B**) The general layout of the result page, showing a summary of information for each entry as well as query keywords, number of total hits, complete search fields for all columns and links (PDB column) to single results page.

In order to access the data, a query/search has to be performed. This can be done either by selecting the ‘Query’ page from the navigation bar or by clicking the ‘Submit Query’ button present on the ‘Home’ page. On the ‘Query’ page, the user has nine different options to search the database (Figure 1A and Supplementary Table S1). We provide on-page guidelines in the form of grey question mark tooltips. Since most users might be unaware of specific IDs, and are more commonly interested in searching for relevant terms or keywords, we implemented a pattern matching algorithm that allows the users to use full keywords (lectin), or partial keyworks (lec*) in some of the query fields (UniProt, Pfam, Enzyme Commission, Organism and Monomer). For example, a keyword query for ‘influenza’ in the organism query field will retrieve 179 entries for several different strains in one simple query.

### Results display

After a query has been submitted by pressing the appropriate ‘Submit’ button, the user will be redirected to either: (i) ‘Multiple Results page if the submitted query returned more than one result (Figure 1B), (ii) ‘General Information’ page (Supplementary Figure S5) if the submitted query returned just one result or (iii) ‘No Result Page’ if no results were found in the database.

On the ‘Multiple Results page (Figure 1B), for each entry obtained as search result, a summary of the available data is displayed. This includes details such as the PDB ID, PubMed ID, UniProt ID, organism name, Pfam ID, Enzyme Classification, PDB Ligand ID(s), name of PDB Ligand ID and availability of affinity values. The query input will be displayed in red (if possible) on the ‘Multiple Results’ page to enable users to easily identify the matched term. Each column of the ‘Multiple Results’ table can be filtered by using the ‘Search’ fields under the headers. Furthermore, the user can download the summary table in .tsv (tab-delimited file) format by selecting the ‘Get TSV’ button (Figure 1B).

In order to access an individual entry, a detailed description is provided in three tabs (namely, ‘General Information’, ‘Ligand Information’ and ‘Mutant Information’), which are described below. Direct links to the ‘Help’ page and to the 3D interactive windows are available on each tab. The website generates intuitive and consistent URLs; hence, users can also bookmark the search pages for easy access.

#### General information tab

Users can click on the PDB ID of an entry obtained as a search result (Figure 1B) and will be directed to the ‘General Information’ page by default (Supplementary Figure S5). This is divided further divided into three sections: (i) information about the crystal structure, (ii) mappings to Pfam domain annotations and UniProt IDs and (iii) an interactive window where the user can inspect different features of the protein–carbohydrate complex, including geometric quality, hydrophobicity and B-factors using informative colour schemes. Users are also able to visually inspect the Pfam-annotated domains, by selecting the Pfam colouring scheme, directly on top of the PDB structure, so allowing the user to identify binding and interface domains.

#### Ligand information tab

The ‘Ligand Information’ tab (Supplementary Figure S6) can be accessed by selecting the appropriate field from the navigation tab. This page is divided into two sections: (i) ligand Information with available biophysical measurements and 3D representation for each ligand and (ii) interactive window where the user can inspect the protein–ligand interface. The first section aims to map individual ligands, rather than whole structures, with affinity values from established databases. The ligand table is user-responsive and linked to the 3D representation window. By selecting the ligand of interest in the table, the 3D representation changes to the selected ligand. Furthermore, all monomer-colouring schemes are conserved and distinct for each monomer throughout the page.

We also provide dedicated 3D representations for all ligands available in a ProCarbDB entry. The user can inspect here the spatial arrangement of a carbohydrate ligand and glycosidic bond order without the added complexity of viewing the entire protein–carbohydrate complex.

#### Mutant information tab

The last tab contains the ‘Mutant Information’ (Supplementary Figure S7) that has been manually curated. These data will be continually updated as part of ongoing curation efforts. The tab is divided into two sections: (i) table of available mutations and (ii) interactive window where the user can inspect the positions of the mutants in the 3D structure of the complex as well as the interactions between the ligand and the wild-type residues. We aim not only to map mutagenesis data from literature but also to identify mutant structures present in ProCarbDB. For example, both 4BLN and 4BLK are PDB IDs present in ProCarbDB. The first structure is identified as wild-type while the second is a K176L mutant. By selecting the corresponding field in the ‘Is mutant in ProCarbDB’ column, users can directly inspect that structure.

Supplementary Table S2 summarizes all the available data as well as the page where it can be accessed.

### 3D interactive windows

**Figure 2.**
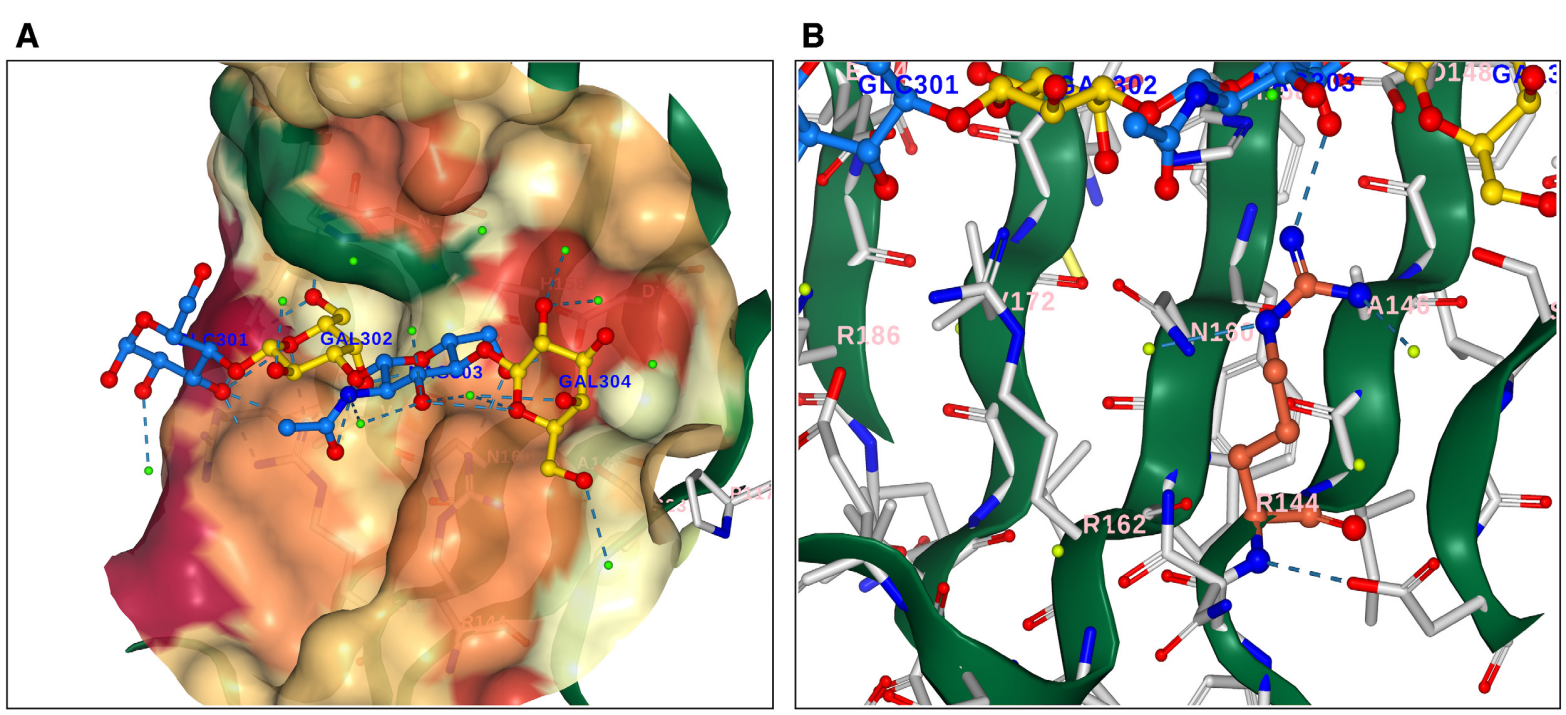
Ligand View (NGL based) for the ligand information page displaying the complete ligand for the PDB ID:4BLN. (**A**) Ligand is displayed as ball-and-stick representation with each distinct PDB residue depicted following SNFG nomenclature: β-D-galactose yellow, α-D-glucose and N-acetyl-D-glucosamine in blue. Water molecules are displayed in light green. Amino acids 4 Å or closer to the ligand are displayed as ball-and-stick representation and coloured in light grey. Contacts are represented in dashed lines. Surface opacity is set to 60%, and coloured based on hydrophobicity (green, for hydrophobic residue, to red, for hydrophilic residues). (**B**) In-depth analysis of the binding pocket. ARG144 was selected and it is displayed in dark orange. In light grey are depicted protein residues. At the top, the same ligand observed in (A) is present, with the same colouring scheme. Only interactions between ARG144 and any other molecule are displayed. Surface opacity is 0%.

3D rendering of macromolecules is imperative for understanding their biological function. Based on our curated data, we are able to calculate and display particularities of the entire structure such as hydrophobicity, secondary structure and Pfam domains. We are also able to map the interface formed by the protein and the complete ligand (Figure 2A). Furthermore, users can have an in-depth analysis of the binding pocket by selecting from the ‘For Mutagenesis’ panel (Figure 2B) any residue of interest 4Å or closer to the ligand. For ProCarbDB entries that are linked with mutation data, we provide a 3D spatial representation of those mutations. In order to maintain consistency and reproducibility, we aimed to keep colouring schemes and definitions as implemented in the PDB.

### Binding affinities

We annotated the complexes present in ProCarbDB with experimentally determined binding data by using already established databases such as MOAD and PDBbind. We retrieved 756 affinity values from MOAD (14) and 626 from PDBbind (13), with an overlap of 415 entries, ultimately generating a collection of 967 complexes with experimentally measured binding affinities. We also checked the values for complexes reporting affinities in both databases and we found out that ∼9% of values do not match. As an example, PDB ID: 5TPC has a *K*_d_ value of 0.3 mM according to MOAD and a *K*_d_ value of 1 mM according to PDBbind. Furthermore, there are many inconsistencies with matching the correct ligand and affinity value. For example, PDB ID: 4D4U has four different affinity values, two of which are for the same ligand on MOAD. This might be in part due to the fact that the authors of the structure could not fully identify the complete ligand (LewisY tetrasaccharide) in all the binding pockets.

An example where the ligand is not properly identified is 4 × 0Z; PDBbind reports a ligand formed by four monosaccharides while the actual ligand is GM1 ganglioside, which contains five monosaccharides. These small inaccuracies in publicly available repositories are due to have major downstream effects on algorithms using their datasets as training sets. For this reason, we tried to solve these inconsistencies, or at least flag them and make it visible to the user in ProCarbDB.

### Data statistics

#### Based on protein partner

**Figure 3.**
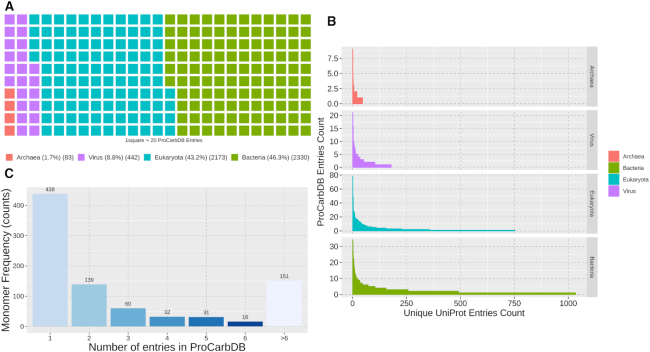
ProCarbDB entries statistics. (**A**) ProCarbDB Phylogenetic Kingdom Distribution. (**B**) UniProt ID Frequency in ProCarbDB per Kingdom. If an UniProt ID is present twice in the same PDB structure, we only count it once in order to normalize the data for homo-oligomers, hetero-oligomers and asymmetric unit protomer duplication. (**C**) Monomer Frequency in ProCarbDB. If a monomer is present twice in the same PDB structure, we only count it once.

We mapped ProCarbDB entries to their kingdom (taxonomy) and identified Bacteria (46.3%) as the most dominant followed by eukaryota (43.2%), viruses (8.8%) and archaea (1.7%) (Figure 3A). Next, we divided the UniProt IDs based on kingdom and counted the number of entries each UniProt ID has in ProCarbDB (Figure 3B). Most UniProt IDs in ProCarbDB (82%) are present in three or less entries. This shows that the data in ProCarbDB are diverse with respect to the UniProt ID distribution. However, it is clear that UniProt IDs from bacteria and eukaryota are dominant in ProCarbDB. The most frequent UniProt ID present in ProCarbDB is ‘P16442’, encoding for histo-blood group ABO system transferase (eukaryota), with 78 entries, followed by ‘P00636’, encoding for fructose-1,6-bisphosphatase 1 protein (eukaryota), with 52 entries (Supplementary Table S3).

To further investigate the redundancy of sequences present in ProCarbDB, we used the CD-Hit (34) software that clusters sequences based on identity, and found that, for a total of 5242 ProCarbDB sequences, CD-Hit identifies 2018 distinct clusters at 90% sequence identity, and 1805 distinct clusters at 70% sequence identity.

#### Based on ligand

Monomers were divided, based on PDB Ligand IDs, into three classes: saccharide (405, 46.7%), glycoconjugate (316, 36.4%) and non-polymers (146, 11.9%). While saccharides contain only sugar rings, monosaccharides or oligosaccharides, glycoconjugate monomers contain at least one non-saccharide moiety, for example ‘UPG’ (uridine-5′-diphospate-glucose) includes uridine.

The complete ligands, comprised of one or more of the above-mentioned monomers, were separated into two classes: saccharide ligands (827, 58.5%) and glycoconjugate ligands (587, 41.5%). We observed that most protein–ligand complexes in ProCarbDB comprised only saccharide moieties (3911/5242), while the rest contain glycoconjugates (1426/5242). There is an overlap of 85 entries that are in both ligand classes due to entries having multiple ligands present in the PDB. In order to ensure that ligand data are also diverse, we counted the number of ProCarbDB entries for each monomer (Figure 3C). Most monomers (73%) in ProCarbDB are present in three or less entries. The most frequent monomers, based on RCSB PDB nomenclature, are GAL, encoding for β-d-galactose, with 818 entries (Supplementary Table S4), followed by, NAG, encoding for N-acetyl-glucosamine, with 621.

Currently ProCarbDB hosts more than 5200 true protein–carbohydrate complexes related to over 2416 PubMed Articles (Table 1). There are 2014 distinct UniProt IDs and 754 distinct Pfam domains.

**Table 1.** Overview of data represented in ProCarbDB

Property	Frequency
Distinct PDB IDs	5242
Distinct UniProt IDs*	2014
Distinct Pfam IDs*	754
Distinct monomers	867
PDB IDs with affinity values	967
PubMed Articles	2416

*For some PDB entries UniProt and/or Pfam mapping was not possible.

## DISCUSSION

While analysis of experimental structures can provide powerful insights into understanding protein function and mechanism of action, this has not been exploited to its full potential for protein–carbohydrate complexes. Carbohydrates are one of the most complex classes of biomolecules from both structural and functional points of view. Thus, the characterization of recognition patterns for carbohydrate-binding proteins is challenging. A repository of high-quality structural and functional data, including the full carbohydrate ligand structures, removing covalently bound structures (post-translational modifications) and displaying the crystal complex in an interactive way will facilitate advancement of the field.

To our knowledge, ProCarbDB is the first repository that is able to retrieve complete ligands via simple queries. We generate and display, in a user-friendly way, not only the interactions between the ligand and its environment, but also the non-allosteric interactions that might be responsible for the binding. The user is able to access 3D interactive windows in a standardized fashion, based on PDB architecture, in order to compare results.

Furthermore, we also attributed functional information, in the form of biophysical measurements. To date, we have linked 18.4% (967) of ProCarbDB entries with at least one experimentally measured binding affinity. We identified and corrected, to the best of our capability, several under-documented issues with currently available databases such as incorrect affinity values and ligands wrongly identified as biologically active. To provide a complete panel of information, we mapped each entry to UniProt, Pfam and NCBI databases. Current efforts are directed towards gathering further mutagenesis information using manual curation, which could not be directly obtained from the external databases.

We believe that ProCarbDB will have a significant impact on the field. Firstly, experimental scientists studying protein–carbohydrate complexes will be able to query ProCarbDB to check whether the protein: (i) has been previously characterized biophysically; (ii) has identified homologs or (iii) has known ligands, in which case they can inspect in depth the protein–carbohydrate interfaces. Secondly, computational scientists will have a comprehensive and refined set of coordinates defining the structures of protein–carbohydrate interfaces as well as a benchmark dataset to train machine-learning algorithms.

ProCarbDB will be an invaluable resource for the understanding and modification of carbohydrate-binding sites and will facilitate the development of new computational tools to analyse these interactions and develop prediction algorithms.

## Supplementary Material

gkz860_Supplemental_File

## Notes

Present address: Sony Malhotra, Birkbeck, Malet Street, University of London, WC1E 7HX, UK.

## References

[1] Ambrosi M., Cameron N.R., Davis B.G. Lectins: tools for the molecular understanding of the glycocode. Org. Biomol. Chem. 2005; 3:1593–1608. 15858635 10.1039/b414350g

[2] Onuma Y., Tateno H., Tsuji S., Hirabayashi J., Ito Y., Asashima M. A lectin-based glycomic approach to identify characteristic features of xenopus embryogenesis. PLoS One. 2013; 8:e56581. 23457585 10.1371/journal.pone.0056581 PMC3572943

[3] Maverakis E., Kim K., Shimoda M., Gershwin M.E., Patel F., Wilken R., Raychaudhuri S., Ruhaak L.R., Lebrilla C.B. Glycans in the immune system and the altered glycan theory of autoimmunity: a critical review. J. Autoimmun. 2015; 57:1–13. 25578468 10.1016/j.jaut.2014.12.002 PMC4340844

[4] Hauri H.-P., Nufer O., Breuza L., Tekaya H.B., Liang L. Lectins and protein traffic early in the secretory pathway. Biochem. Soc. Symp. 2002; 69:73–82. 10.1042/bss0690073 12655775

[5] Zuverink M., Barbieri J.T. Protein toxins that utilize gangliosides as host receptors. Prog. Mol. Biol. Transl. Sci. 2018; 156:325–354. 29747819 10.1016/bs.pmbts.2017.11.010 PMC6243200

[6] Chen L., Li F. Structural analysis of the evolutionary origins of influenza virus hemagglutinin and other viral lectins. J. Virol. 2013; 87:4118–4120. 23365425 10.1128/JVI.03476-12 PMC3624229

[7] Burley S.K., Berman H.M., Bhikadiya C., Bi C., Chen L., Di Costanzo L., Christie C., Dalenberg K., Duarte J.M., Dutta S. et al. RCSB Protein Data Bank: biological macromolecular structures enabling research and education in fundamental biology, biomedicine, biotechnology and energy. Nucleic Acids Res. 2019; 47:D464–D474. 30357411 10.1093/nar/gky1004 PMC6324064

[8] Lütteke T., Frank M., von der Lieth C.-W. Data mining the protein data bank: automatic detection and assignment of carbohydrate structures. Carbohydr. Res. 2004; 339:1015–1020. 15010309 10.1016/j.carres.2003.09.038

[9] Schrödinger LLC The PyMOL Molecular Graphics System, Version 2.0. 1 October 2019, date last accessed https://pymol.org/2/.

[10] Pettersen E.F., Goddard T.D., Huang C.C., Couch G.S., Greenblatt D.M., Meng E.C., Ferrin T.E. UCSF Chimera–a visualization system for exploratory research and analysis. J. Comput. Chem. 2004; 25:1605–1612. 15264254 10.1002/jcc.20084

[11] Lütteke T., von der Lieth C.-W. pdb-care (PDB carbohydrate residue check): a program to support annotation of complex carbohydrate structures in PDB files. BMC Bioinform. 2004; 5:69. 10.1186/1471-2105-5-69 PMC441419 15180909

[12] Pires D.E. V, Blundell T.L., Ascher D.B. Platinum: a database of experimentally measured effects of mutations on structurally defined protein-ligand complexes. Nucleic Acids Res. 2014; 43:387–391. 10.1093/nar/gku966 PMC4384026 25324307

[13] Liu Z., Su M., Han L., Liu J., Yang Q., Li Y., Wang R. Forging the basis for developing protein–ligand interaction scoring functions. Acc. Chem. Res. 2017; 50:302–309. 28182403 10.1021/acs.accounts.6b00491

[14] Ahmed A., Smith R.D., Clark J.J., Dunbar J.B., Carlson H.A. Recent improvements to Binding MOAD: a resource for protein–ligand binding affinities and structures. Nucleic Acids Res. 2015; 43:D465–D469. 25378330 10.1093/nar/gku1088 PMC4383918

[15] Yowler B.C., Schengrund C.-L. Botulinum Neurotoxin A changes conformation upon binding to ganglioside GT1b. Biochemistry. 2004; 43:9725–9731. 15274627 10.1021/bi0494673

[16] Benson M.A., Fu Z., Kim J.-J.P., Baldwin M.R. Unique ganglioside recognition strategies for clostridial neurotoxins. J. Biol. Chem. 2011; 286:34015–34022. 21849494 10.1074/jbc.M111.272054 PMC3190786

[17] Hamark C., Berntsson R.P.-A., Masuyer G., Henriksson L.M., Gustafsson R., Stenmark P., Widmalm G. Glycans confer specificity to the recognition of ganglioside receptors by botulinum Neurotoxin A. J. Am. Chem. Soc. 2017; 139:218–230. 27958736 10.1021/jacs.6b09534

[18] Pires D.E. V, Blundell T.L., Ascher D.B. mCSM-lig: quantifying the effects of mutations on protein-small molecule affinity in genetic disease and emergence of drug resistance. Sci. Rep. 2016; 6:29575. 27384129 10.1038/srep29575 PMC4935856

[19] Banno M., Komiyama Y., Cao W., Oku Y., Ueki K., Sumikoshi K., Nakamura S., Terada T., Shimizu K. Development of a sugar-binding residue prediction system from protein sequences using support vector machine. Comput. Biol. Chem. 2017; 66:36–43. 27889654 10.1016/j.compbiolchem.2016.10.009

[20] Stepniewska-Dziubinska M.M., Zielenkiewicz P., Siedlecki P. Development and evaluation of a deep learning model for protein-ligand binding affinity prediction. Bioinformatics. 2018; 34:3666–3674. 29757353 10.1093/bioinformatics/bty374 PMC6198856

[21] Bonnardel F., Mariethoz J., Salentin S., Robin X., Schroeder M., Perez S., Lisacek F.D.S., Imberty A. Unilectin3d, a database of carbohydrate binding proteins with curated information on 3D structures and interacting ligands. Nucleic Acids Res. 2019; 47:D1236–D1244. 30239928 10.1093/nar/gky832 PMC6323968

[22] Thieker D.F., Hadden J.A., Schulten K., Woods R.J. 3D implementation of the symbol nomenclature for graphical representation of glycans. Glycobiology. 2016; 26:786–787. 27514939 10.1093/glycob/cww076 PMC5018049

[23] McNaught A.D. Nomenclature of carbohydrates (recommendations 1996). Adv. Carbohydr. Chem. Biochem. 1997; 52:43–177. 9218333

[24] Lombard V., Golaconda Ramulu H., Drula E., Coutinho P.M., Henrissat B. The carbohydrate-active enzymes database (CAZy) in 2013. Nucleic Acids Res. 2014; 42:D490–D495. 24270786 10.1093/nar/gkt1178 PMC3965031

[25] Tiemeyer M., Aoki K., Paulson J., Cummings R.D., York W.S., Karlsson N.G., Lisacek F., Packer N.H., Campbell M.P., Aoki N.P. et al. GlyTouCan: An accessible glycan structure repository. Glycobiology. 2017; 27:915–919. 28922742 10.1093/glycob/cwx066 PMC5881658

[26] Choudhary P., Nagar R., Singh V., Bhat A.H., Sharma Y., Rao A. ProGlycProt V2.0, a repository of experimentally validated glycoproteins and protein glycosyltransferases of prokaryotes. Glycobiology. 2019; 29:461–468. 30835791 10.1093/glycob/cwz013

[27] Toukach P. V., Egorova K.S. Carbohydrate structure database merged from bacterial, archaeal, plant and fungal parts. Nucleic Acids Res. 2016; 44:D1229–D1236. 26286194 10.1093/nar/gkv840 PMC4702937

[28] Pérez S., Sarkar A., Rivet A., Breton C., Imberty A. Glyco3D: a portal for structural glycosciences. Methods Mol. Biol. 2015; 1273:241–258. 25753716 10.1007/978-1-4939-2343-4_18

[29] UniProt Consortium UniProt: a worldwide hub of protein knowledge. Nucleic Acids Res. 2019; 47:D506–D515. 30395287 10.1093/nar/gky1049 PMC6323992

[30] Bairoch A. The ENZYME database in 2000. Nucleic Acids Res. 2000; 28:304–305. 10592255 10.1093/nar/28.1.304 PMC102465

[31] Bohne-Lang A., Lang E., Förster T., von der Lieth C.W. LINUCS: linear notation for unique description of carbohydrate sequences. Carbohydr. Res. 2001; 336:1–11. 11675023 10.1016/s0008-6215(01)00230-0

[32] El-Gebali S., Mistry J., Bateman A., Eddy S.R., Luciani A., Potter S.C., Qureshi M., Richardson L.J., Salazar G.A., Smart A. et al. The Pfam protein families database in 2019. Nucleic Acids Res. 2019; 47:D427–D432. 30357350 10.1093/nar/gky995 PMC6324024

[33] Rose A.S., Bradley A.R., Valasatava Y., Duarte J.M., Prlic A., Rose P.W. NGL viewer: web-based molecular graphics for large complexes. Bioinformatics. 2018; 34:3755–3758. 29850778 10.1093/bioinformatics/bty419 PMC6198858

[34] Fu L., Niu B., Zhu Z., Wu S., Li W. CD-HIT: accelerated for clustering the next-generation sequencing data. Bioinformatics. 2012; 28:3150–3152. 23060610 10.1093/bioinformatics/bts565 PMC3516142

