# Supplementary material for: ProCarbDB: a database of carbohydrate-binding proteins
Source: Nucleic Acids Res. 2019 Oct 10;48(D1):D368–75. doi: 10.1093/nar/gkz860 (PMC6943041; doi:10.1093/nar/gkz860)
Supplement: gkz860_Supplemental_File [file gkz860_supplemental_file.docx]

**SUPPLEMENTARY MATERIAL**

**ProCarbDB: A database of carbohydrate binding proteins**

Liviu Copoiu^1^, Pedro H.M. Torres^1^, David B. Ascher^1,2^, Tom L. Blundell^1^*, Sony Malhotra^1,3^*

^1^Department of Biochemistry, University of Cambridge, Tennis Court Road, Cambridge, UK ^2^Department of Biochemistry, University of Melbourne, Flemington Road, Parkville, Australia ^3^Present address: Birkbeck, Malet Street, University of London, WC1E 7HX, UK

*To whom correspondence should be addressed T.L.B. Tel: +44 1223 333628; Email: [tom@cryst.bioc.cam.ac.uk](mailto:tom@cryst.bioc.cam.ac.uk). Correspondence may also be addressed to S.M. s.malhotra@mail.cryst.bbk.ac.uk.

**
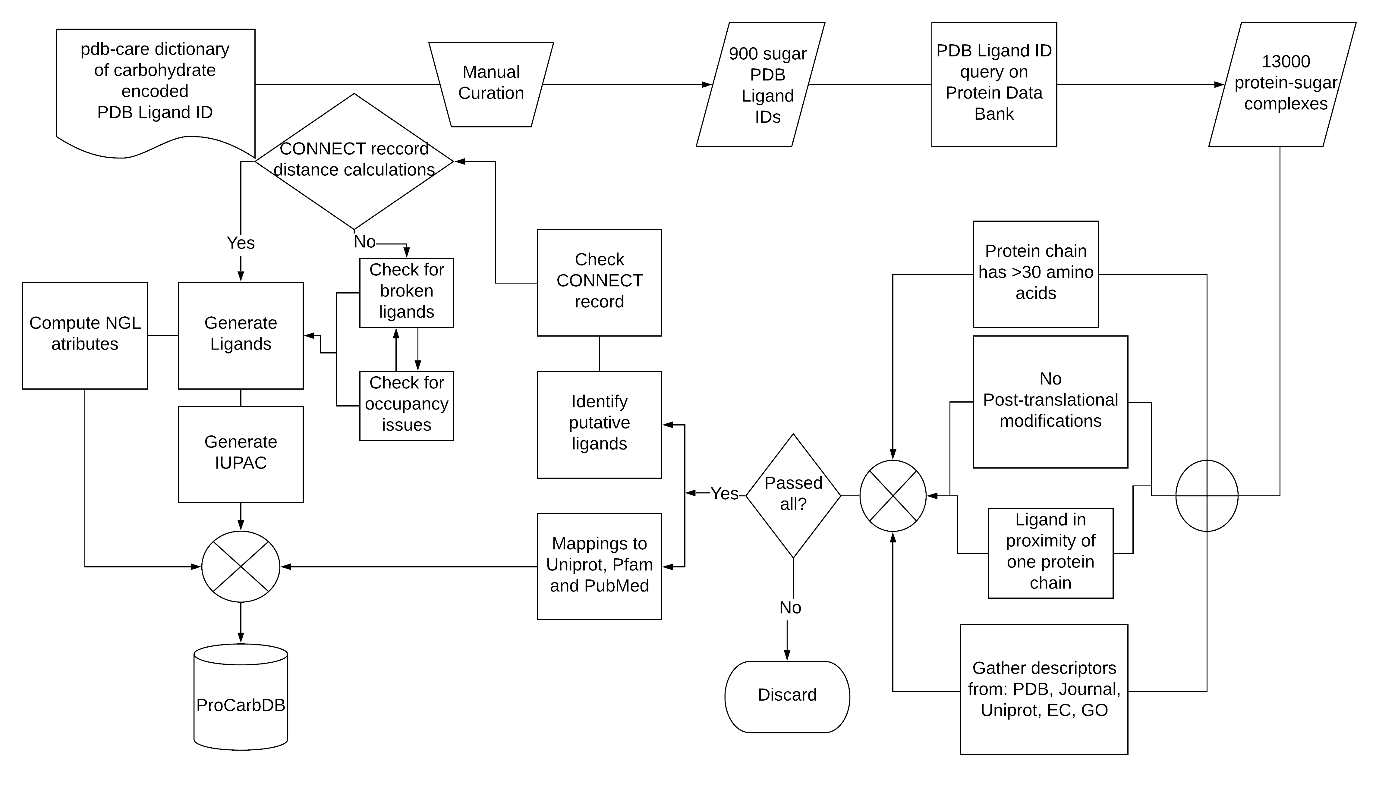
**

**Figure S1:** Flowchart of the pipeline used to access, filter and deposit protein-carbohydrate complexes.


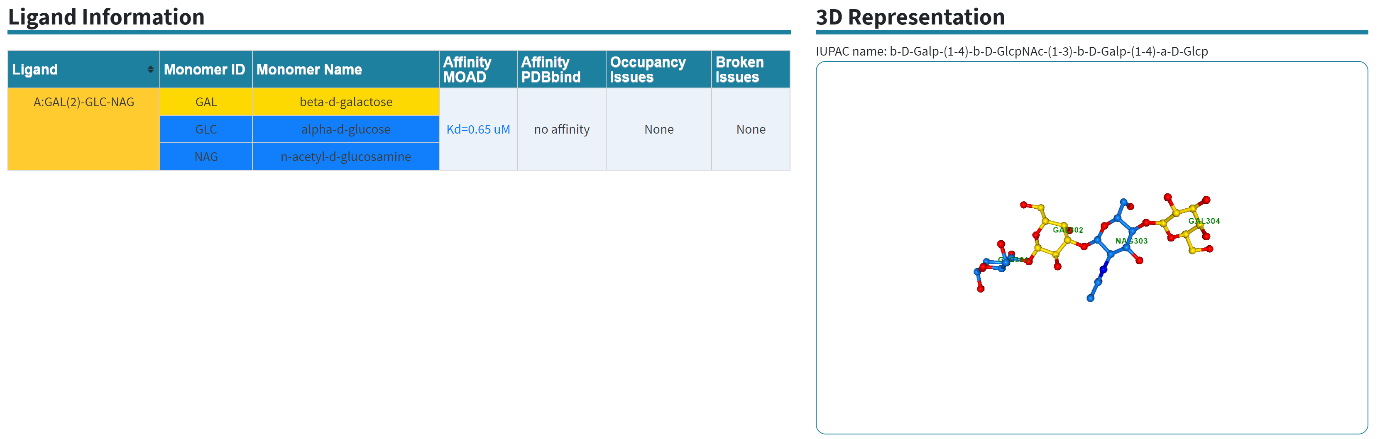


**Figure S2:** Display of complete ligands and matching 3D representation present in ProCarbDB following SNFG nomenclature and displaying the IUPAC name of the selected ligand


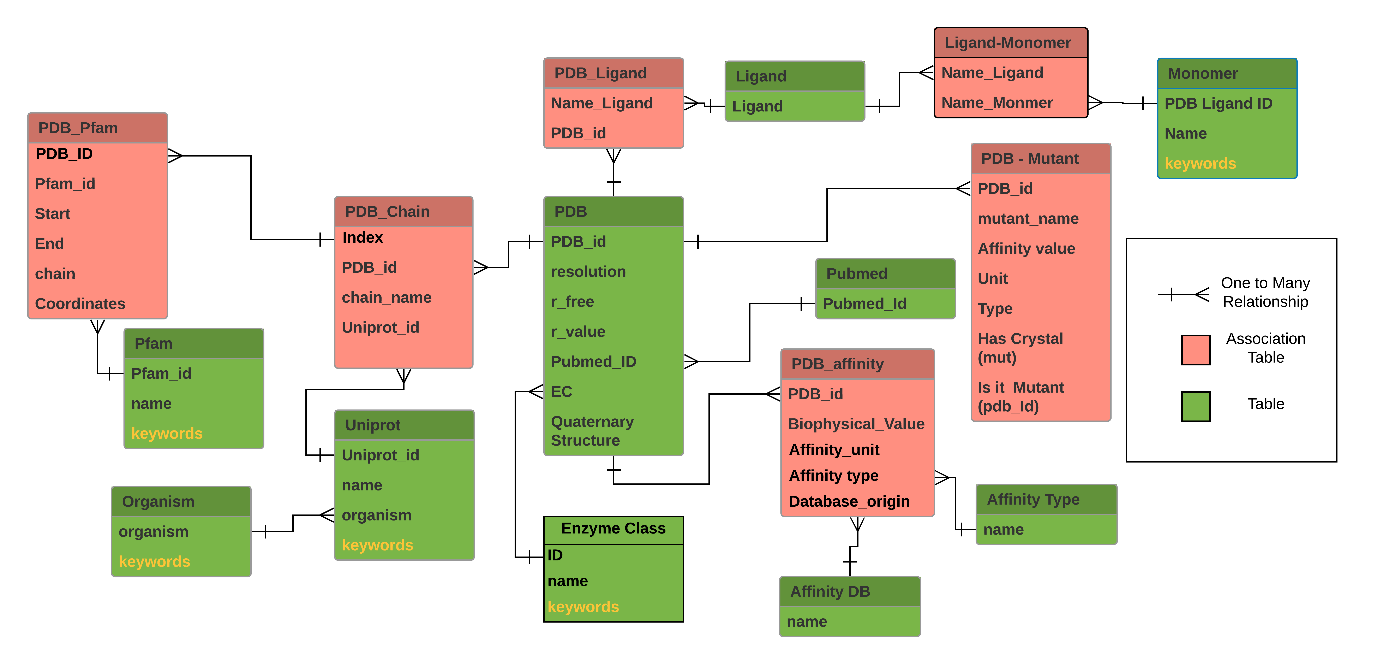


**Figure S3:** ProCarbDB database architecture schema. Each rectangle represents an SQLAlchemy table, Association tables are used to resolve Many-to-Many Relationships.

**
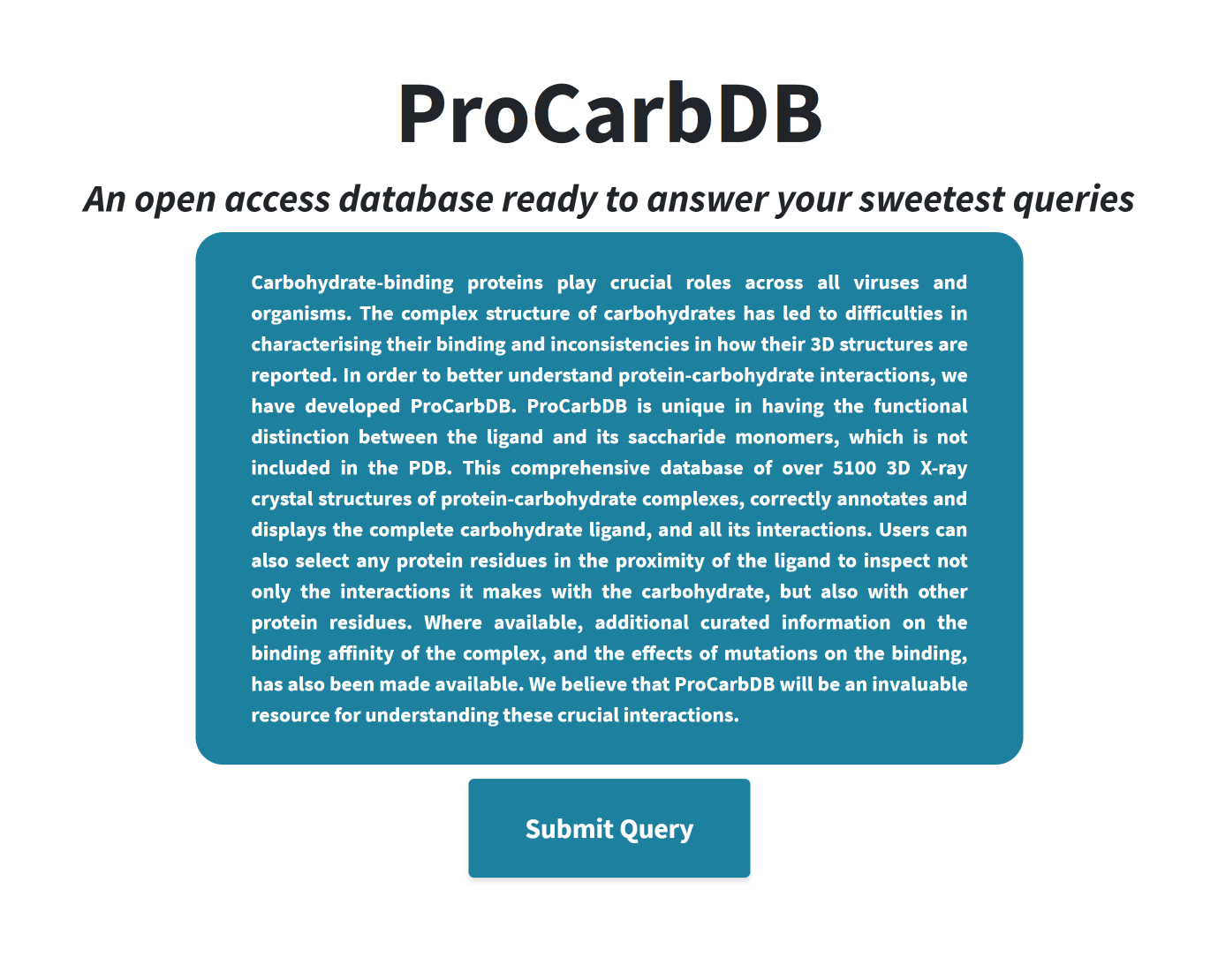
**

**Figure S4:** ‘Home’ page displaying the navigation bar (top) with links to general pages such as: ‘Help’, and ‘Query’ as well as small description (center) of ProCarbDB.


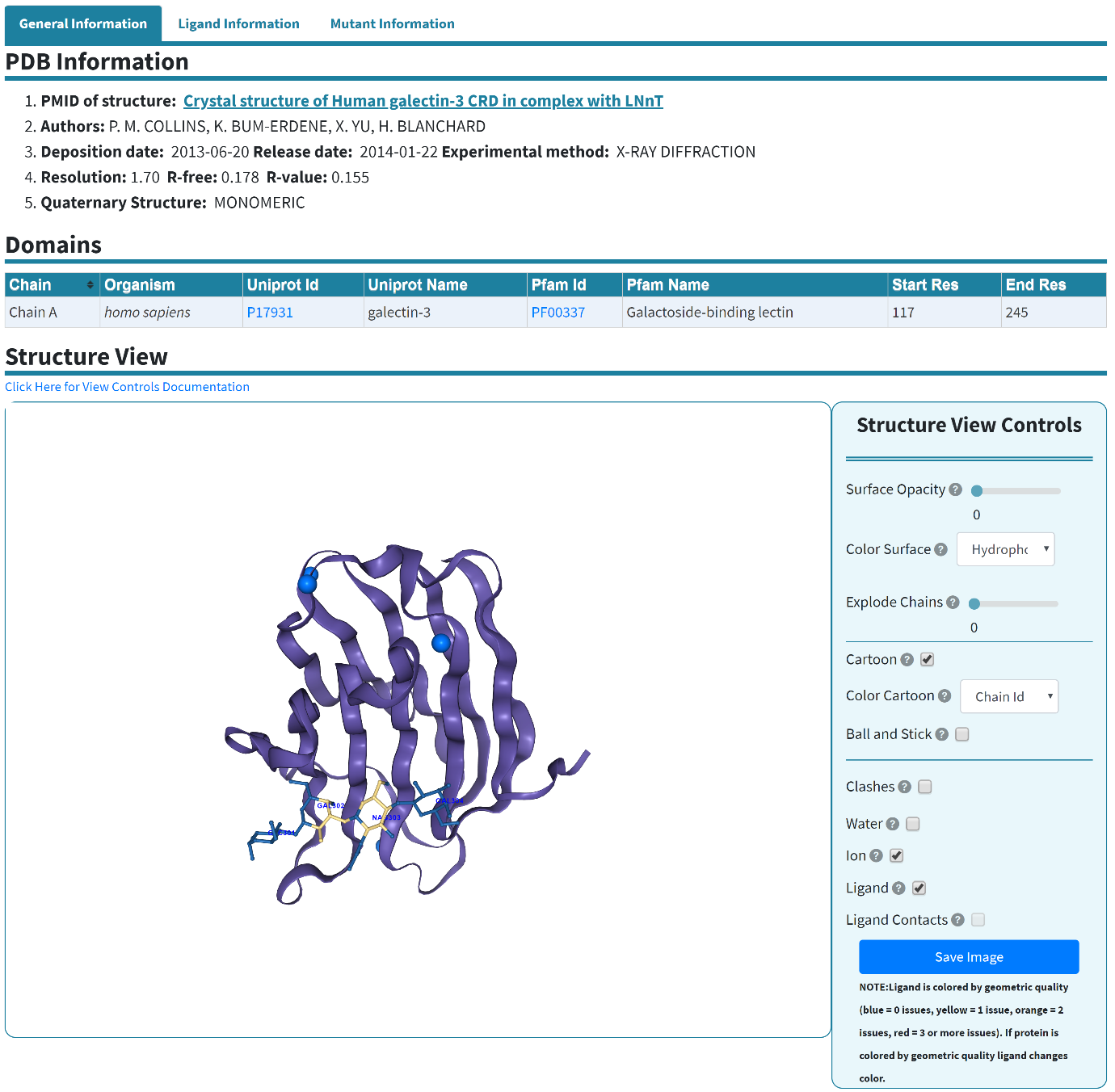


**Figure S5:** ‘General Information’ page for individual results displaying the current(active) page, the 3 available sections and the controls window for Structure View

**
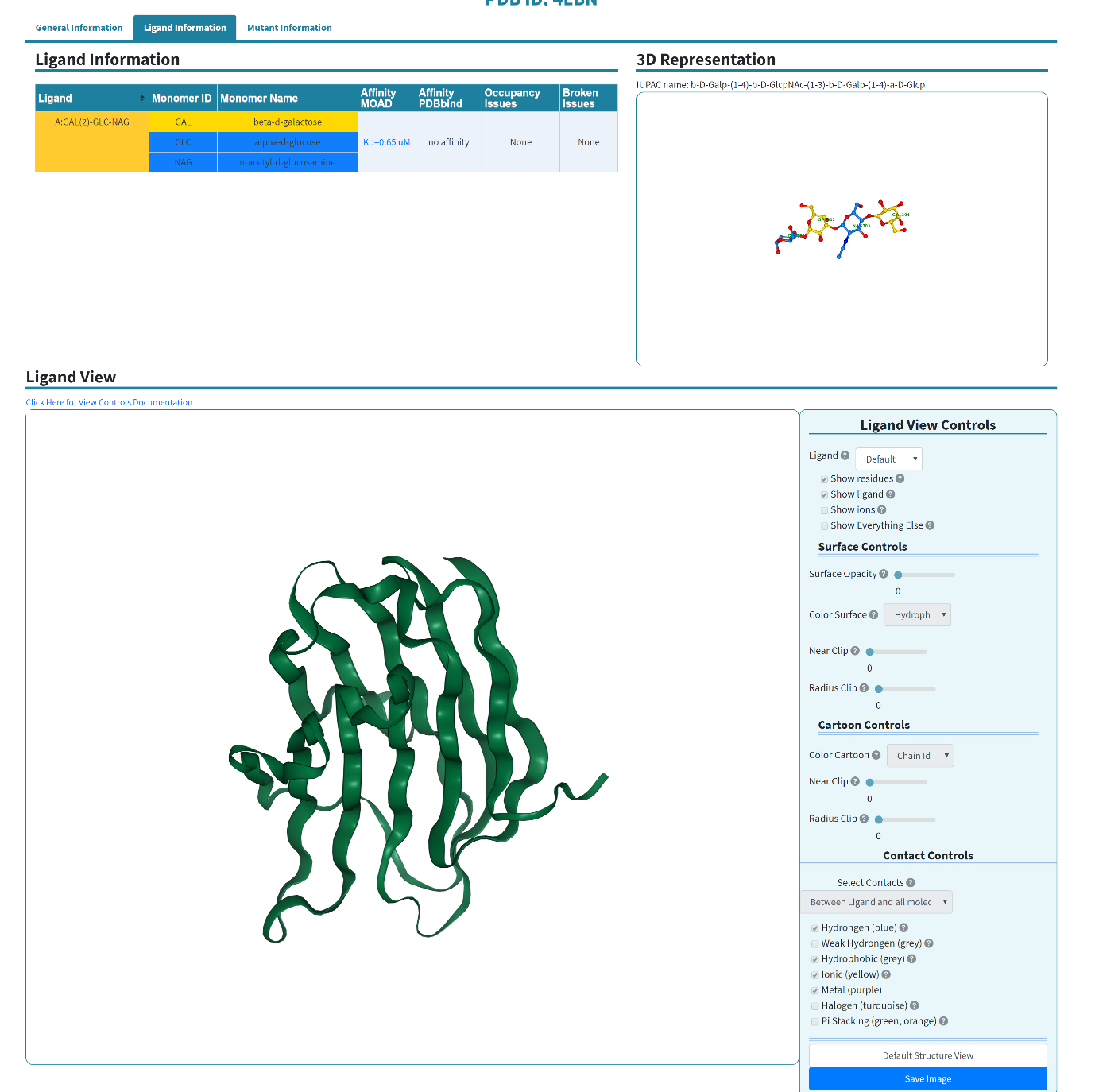
**

**Figure S6:** ‘Ligand Information’ page for individual results displaying the current(active) page, the 2 available sections and the controls window for Structure View


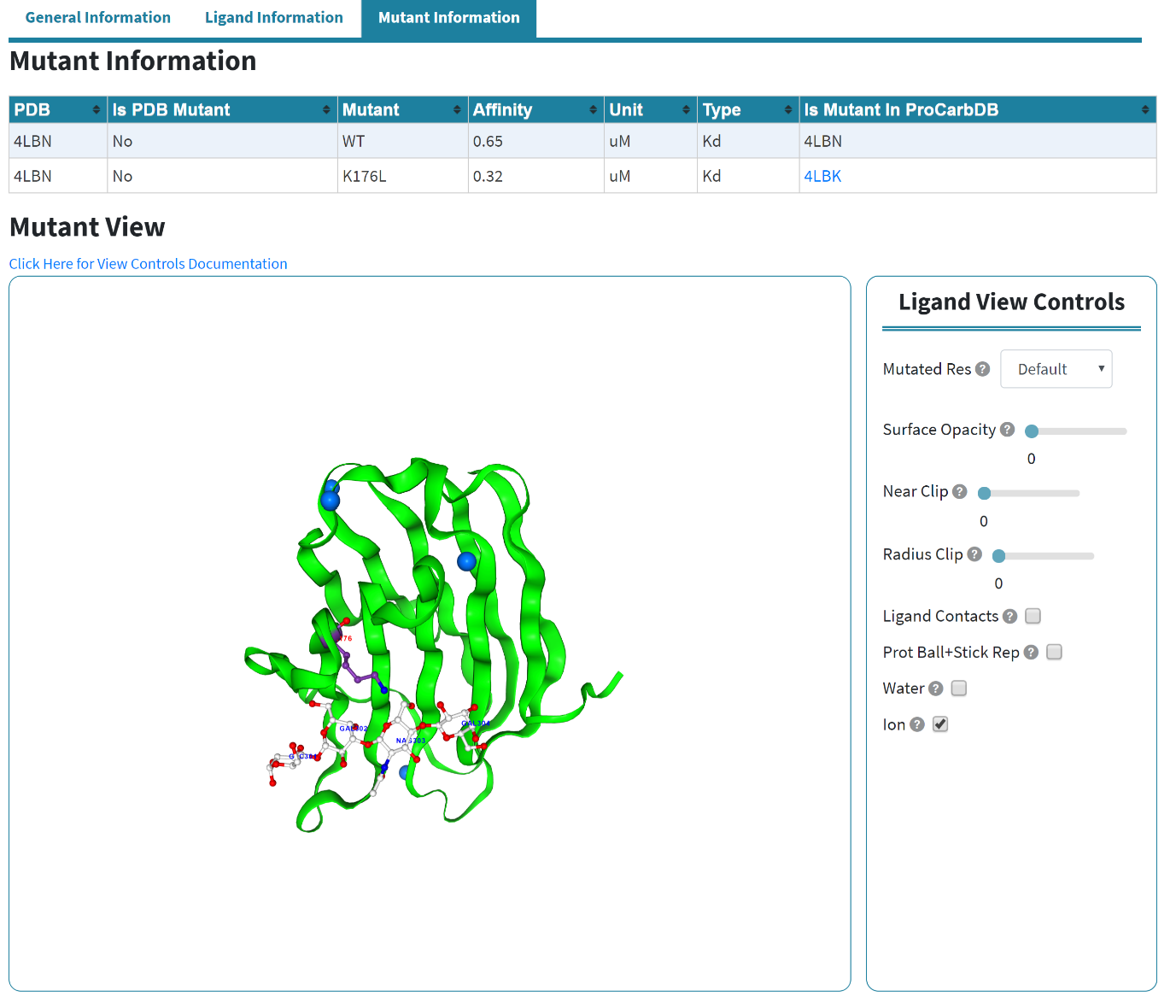


**Figure S7:** ‘Mutant Information’ page for individual results displaying the current(active) page, the 2 available sections and the controls window for Mutant view

**Table S1:** Query modes available in ProCarbDB

Screenshot of ‘Query’ Page displaying query modes as well as the top navigation bar and on top-right corner and a dedicated link towards the ‘Help’ page. For each query type we offer on-page help in form of question mark tooltips.

| Query Modes | Description |
| --- | --- |
| PDB ID | Uses the following regex pattern: ^[0-9][a-zA-Z0-9]{3}$, as explained at rcsb.org |
| UniProt ID | Uses the following regex pattern: ^[OPQ][0-9][A-Z0-9]{3}[0-9]\|[A-NR-Z][0-9]([A-Z][A-Z0-9]{2}[0-9]){1,2}$, as explained at uniprot.org. Keywords and partial term searches are supported for this query type. |
| Pfam ID | Uses the following regex pattern: ^PF\d{5}.\d{1,}\|^PF\d{5}$, as explained at pfamx.org. Keywords and partial term searches are supported for this query type. |
| PDB Ligand ID | Uses the following regex pattern: ^[A-Z0-9]{3}$, as explained at rcsb.org. Keywords and partial term searches are supported for this query type. |
| Enzyme Classification | Uses the following regex pattern: ^[1-7]*. Keywords and partial term searches are supported for this query type. |
| Taxonomy | String type representing the species/strain level. Keywords and partial term searches are supported for this query type. |
| Feature | Most general type of query that allows the user to retrieve ProCarbDB entries that have unique parameters |
| Sequence | Blast search of input over local database of sequences generated from PDB files. Parameters(E-value = 0.0001, coverage = 85%, identity = 85%) |
| Affinity | The user can select from 10 types of affinities (such as dissociation constant). A more in depth version of the Feature query |

**Table S2:** Data availability on ProCarbDB.

| Data | Multiple Hits Page | General Information Page | Ligand Information Page | Mutant Information Page |
| --- | --- | --- | --- | --- |
| PDB ID | ✓ | ✓ | ✓ | ✓ |
| UniProt ID(s) | ✓ | ✓ |  |  |
| Pfam ID(s) | ✓ | ✓ |  |  |
| PMID | ✓ | ✓ |  |  |
| Pfam Name | ✓ | ✓ |  |  |
| Organism | ✓ | ✓ |  |  |
| Ligands |  |  | ✓ |  |
| PDB Ligand ID(s) | ✓ |  | ✓ |  |
| IUPAC Name | ✓ |  | ✓ |  |
| Affinity Data | ✓ |  | ✓ | ✓ |
| 3D Interactive Windows |  | ✓ | ✓ | ✓ |

**Table S3**: UniProt name frequency

| Nr | UniProt ID and name | Counts |
| --- | --- | --- |
| 1 | P16442 :histo-blood group abo system transferase | 78 |
| 2 | P00636: fructose-1,6-bisphosphatase 1 | 52 |
| 3 | P00489: glycogen phosphorylase, muscle form | 49 |
| 4 | P08191: type 1 fimbrin d-mannose specific adhesin | 34 |
| 5 | P0AEX9: maltose/maltodextrin-binding periplasmic protein | 32 |
| 7 | Q8GSD2: lectin | 28 |
| 8 | P24627: lactotransferrin | 28 |
| 9 | P01887: beta-2-microglobulin | 28 |
| 10 | P11609: antigen-presenting glycoprotein cd1d1 | 28 |
| 12 | P17931: galectin-3 | 27 |
| 13 | P00722: beta-galactosidase | 27 |
| 14 | P00698: lysozyme c | 25 |
| 13 | P18670: agglutinin alpha chain | 24 |
| 14 | P9WFX5: anthranilate phosphoribosyltransferase | 23 |
| 15 | P43379: cyclomaltodextrin glucanotransferase | 22 |
| 16 | Q9HYN5: fucose-binding lectin pa-iil | 22 |
| 17 | Q9AYY6: tail spike protein | 21 |
| 18 | P18673: agglutinin beta-3 chain | 20 |
| 19 | P35247: pulmonary surfactant-associated protein d | 19 |
| 20 | P04746: pancreatic alpha-amylase | 19 |

**Table S4**: PDB Ligand frequency

| Nr | Name of Ligand | Counts |
| --- | --- | --- |
| 1 | β-D-Galactose | 818 |
| 2 | N-Acetyl-D-Glucosamine | 621 |
| 3 | β-D-Glucose | 599 |
| 4 | α-D-Glucose | 544 |
| 5 | Sialic Acid | 362 |
| 6 | α-D-Mannose | 255 |
| 7 | α-L-Fucose | 224 |
| 8 | α -D-Galactose | 179 |
| 9 | β-D-Xylopyranose | 154 |
| 10 | β-D-Mannose | 125 |
| 11 | 2-(Acetylamono)-2-Deoxy-A-D-Glucopyranose | 120 |
| 12 | N-Acetyl-2-Deoxy-2-Amino-Galactose | 110 |
| 13 | N-Acetyl-D-Galactosamine | 107 |
| 14 | Fructose-6-Phospahte | 92 |
| 15 | Uridine-5’-Diphospate-Glucose | 87 |
| 16 | Maltose | 84 |
| 17 | Xylopyranose | 76 |
| 18 | β -Lactose | 74 |
| 19 | Uridine-Diphosphate-N-Acetylglucosamine | 66 |
| 20 | Cellobiose | 63 |
